# Whole genome sequencing and comparative genomics of *Mycobacterium orygis* isolated from different animal hosts to identify specific diagnostic markers

**DOI:** 10.3389/fcimb.2023.1302393

**Published:** 2023-12-22

**Authors:** Kumaragurubaran Karthik, Saraswathi Subramanian, Michael Vinoli Priyadharshini, Ayyaru Jawahar, Subbaiyan Anbazhagan, Ramaiyan Selvaraju Kathiravan, Prasad Thomas, Ramasamy Parthiban Aravindh Babu, Krishnaswamy Gopalan Tirumurugaan, Gopal Dhinakar Raj

**Affiliations:** ^1^Department of Veterinary Microbiology, Veterinary College and Research Institute, Tamil Nadu Veterinary and Animal Sciences University, Udumalpet, India; ^2^Translational Research Platform for Veterinary Biologicals, Tamil Nadu Veterinary and Animal Sciences University (TANUVAS), Chennai, India; ^3^Indian Council of Medical Research (ICMR)-National Animal Resource Facility for Biomedical Research, Hyderabad, Telangana, India; ^4^Division of Bacteriology and Mycology, Indian Council of Agricultural Research (ICAR)- India Veterinary Research Institute, Bareilly, Uttar Pradesh, India; ^5^Department of Animal Biotechnology, Madras Veterinary College, Tamil Nadu Veterinary and Animal Sciences University (TANUVAS), Chennai, India

**Keywords:** *Mycobacterium orygis*, buffalo, sambar deer, India, comparative genomics, region of difference, SNP

## Abstract

**Introduction:**

*Mycobacterium orygis*, a member of MTBC has been identified in higher numbers in the recent years from animals of South Asia. Comparative genomics of this important zoonotic pathogen is not available which can provide data on the molecular difference between other MTBC members. Hence, the present study was carried out to isolate, whole genome sequence *M. orygis* from different animal species (cattle, buffalo and deer) and to identify molecular marker for the differentiation of *M. orygis* from other MTBC members.

**Methods:**

Isolation and whole genome sequencing of *M. orygis* was carried out for 9 samples (4 cattle, 4 deer and 1 buffalo) died due to tuberculosis. Comparative genomics employing 53 genomes (44 from database and 9 newly sequenced) was performed to identify SNPs, spoligotype, pangenome structure, and region of difference.

**Results:**

*M. orygis* was isolated from water buffalo and sambar deer which is the first of its kind report worldwide. Comparative pangenomics of all *M. orygis* strains worldwide (n= 53) showed a closed pangenome structure which is also reported for the first time. Pairwise SNP between TANUVAS_2, TANUVAS_4, TANUVAS_5, TANUVAS_7 and NIRTAH144 was less than 15 indicating that the same *M. orygis* strain may be the cause for infection. Region of difference prediction showed absence of RD7, RD8, RD9, RD10, RD12, RD301, RD315 in all the *M. orygis* analyzed. SNPs in virulence gene, PE35 was found to be unique to *M. orygis* which can be used as marker for identification.

**Conclusion:**

The present study is yet another supportive evidence that *M. orygis* is more prevalent among animals in South Asia and the zoonotic potential of this organism needs to be evaluated.

## 1 Introduction

Members of the *Mycobacterium tuberculosis* complex (MTBC) cause tuberculosis in humans and various animal species. *M. bovis* was traditionally thought to be the major infectious zoonotic pathogen causing animal tuberculosis worldwide. Later, it was reported that another member of MTBC, *M. orygis*, was found in higher numbers in cases of animal tuberculosis in South Asia (Thapa et al., 2022). *M. orygis* has been isolated from different animal species such as cattle, bison, spotted deer, rhesus monkey, free-ranging rhinoceros, black buck, and African buffalo (Thapa et al., 2015; Thapa et al., 2016; Rahim et al., 2017; Refaya et al., 2019; Sharma et al., 2023). Human cases of tuberculosis due to *M. orygis* have been documented in New Zealand (Dawson et al., 2012), Australia (Lavender et al., 2013), the United States of America (Marcos et al., 2017), the United Kingdom (Lipworth et al., 2019), Norway (Eldholm et al., 2021), the Netherlands, and recently India (Sumanth et al., 2023). There is growing evidence that the number of animal tuberculosis cases in India caused by *M. orygis* is higher than cases caused by other MTBC members.

The biology, epidemiology, and genomics of *M. orygis* are poorly understood. There is a difference of opinion among researchers on the primary host of *M. orygis*. It was previously reported that the organism was an animal-adapted MTBC member that mainly affects wild animals (van Ingen et al., 2012), while a recent hypothesis states that cattle are the primary host, and spill-over events can lead to infection in wild animals and humans (Rahim et al., 2017; Brites et al., 2018). The sudden rise in the number of *M. orygis* infections in South Asia may be due to the lack of assays that can clearly discriminate the members of MTBC, leading to incorrect reporting as *M. bovis* or *M. tuberculosis*. Tools such as multilocus sequence typing (MLST), spoligotyping, region of difference (RDs) analysis, and single nucleotide polymorphisms (SNPs) can be used for the discrimination of MTBC members. Recently, RDs specific to *M. orygis* were reported, although only 32 genomes were used for analysis, and further validation was required to use these RDs as specific markers for the differentiation of *M. orygis* (Bespiatykh et al., 2021).

Next-generation sequencing and analysis have provided a platform for clear differentiation of bacterial strains. Recently, a number of reports documented the isolation and whole genome sequencing of *M. orygis* from domestic and captive wild animals (Refaya et al., 2022; Sharma et al., 2023). Still, there is no comprehensive study comprising the isolation and whole genome sequencing (WGS) of *M. orygis* from different animal species. Hence, in the present study, the WGS of *M. orygis* from cattle, water buffalo, sambar deer, and spotted deer was carried out. Comparative genomics of *M. orygis* was carried out in this study using publicly available data to identify a marker for the discrimination of *M. orygis* from other MTBC members.

## 2 Methods

### 2.1 Sample collection

Lung and lymph node samples were collected from nine dead animals (cattle= 4, buffalo= 1, deer= 4) that were suspected as having had tuberculosis (**Table 1**).

**Table 1:** Details of the samples processed for isolation of *Mycobacterium* spp.

S. No.	Sample ID.	Species	Place of collection	Samples collected	Remarks
1	1137	Cattle	Chengalpattu, Tamil Nadu	Lung, lymph nodes	Single intradermal comparative cervical tuberculin (SICCT) test – reactor animal
2	C1	Cattle	Chennai, Tamil Nadu	Lung, lymph nodes	–
3	Deer 3	Sambar deer	Chennai, Tamil Nadu	Lung, lymph nodes	–
4	Deer 4	Sambar deer	Chennai, Tamil Nadu	Lung, lymph nodes	–
5	1046	Cattle	Chengalpattu, Tamil Nadu	Lung, lymph nodes	SICCT – reactor animal
6	JX25	Cattle	Chengalpattu, Tamil Nadu	Lung, lymph nodes	SICCT – reactor animal
7	Deer 1	Deer	Chennai, Tamil Nadu	Lung, lymph nodes	–
8	Deer 5	Spotted deer	Chennai, Tamil Nadu	Lung, lymph nodes	–
9	M44	Buffalo	Chengalpattu, Tamil Nadu	Lung, lymph nodes	SICCT – reactor animal

Cattle and buffalo were maintained in an organized farm in Chengalpattu district, Tamil Nadu, India. Post-mortem examination of dead cattle and buffalo was done to identify the cause of death. It should be mentioned that three cattle and one buffalo were tested to be reactors for tuberculosis by a single intradermal comparative cervical tuberculin (SICCT) test earlier in 2021. Free-ranging deer in Guindy National Park Forest area in Chennai that had died from unknown causes were presented for post-mortem at Madras Veterinary College, Chennai. Post-mortem examination of the dead animals revealed numerous nodules in the lungs and lymph nodes, while one cattle (ID 1046) had numerous nodules in the liver. All the collected samples were processed in the BACTEC MGIT 960 system for isolation of *Mycobacterium* spp. All the samples were initially decontaminated using sodium hydroxide-NALC. After inoculation of the samples, MGIT tubes were incubated in the BACTEC instrument for 45 days before declaring the sample as negative for the presence of *Mycobacterium* spp. MGIT tubes with growth units/turbidity were used for acid-fast staining, and acid-fast positive samples were further used for isolation on an LJ solid medium. Simultaneously, acid-fast positive MGIT cultures were used for molecular confirmation. DNA was extracted from 1.0 ml of BACTEC MGIT culture fluid using a MagGenome Xpress DNA kit following the manufacturer’s instructions. PCR for the amplification of *Mycobacterium tuberculosis* complex (MTBC) was performed using primers targeting the IS*6110* region (Miller et al., 1997). Differentiation among *M. tuberculosis*, *M. bovis*, and *M. orygis* was performed using primers targeting the RD12 region (Duffy et al., 2020).

### 2.2 Whole genome sequencing, assembly, and annotation of *M. orygis*

All of the nine *M. orygis* isolates were used for whole genome sequencing. DNA samples extracted using the MagGenome Xpress DNA kit were submitted to MedGenome Labs Ltd., Bengaluru, Karnataka, for whole genome sequencing using Illumina HiseqX technology. The quality of raw reads were tested with the FastQC tool (http://www.bioinformatics.babraham.ac.uk/projects/fastqc/). For *de novo* genome assembly, Unicycler version 0.4.8.0 was used with default options. Normal bridging mode and spades error correction were used to create error-free genome assembly. Annotation was carried out with Prokka 1.14.5 and the Rapid Annotations using Subsystem Technology (RAST) annotation pipeline (Aziz et al., 2008; Tatusova et al., 2016; Wick et al., 2017). The virulence factors of the *M. orygis* genomes (n=9) were predicted with the VFDB: virulence factor database (Liu B. et al., 2022). Accessibility of the database was carried out with the ABRicate tool available in the Galaxy server (Galaxy Version 1.0.1). For this, a minimum of 75% DNA identity and 80% DNA coverage was used (Seemann, 2016). All the genome generated in this study was submitted to the NCBI SRA database with the bio project number PRJNA785380.

### 2.3 Whole genome phylogeny

The whole genome sequence assemblies of *M. orygis* (n=9) isolated in this study were used along with 45 genome assemblies of *M. orygis* and other MTBC isolates (n=40) retrieved from the NCBI database (**Supplementary Table 1**). All the genome assemblies were aligned using the Reference Sequence Alignment Based Phylogeny Builder (REALPHY) (https://realphy.unibas.ch/realphy/) pipeline employing *M. tuberculosis* H37Rv as the reference genome for the genome alignment (Bertels et al., 2014). Whole genome phylogeny was constructed with the maximum likelihood (ML) method and substitution model (GTR+G). Bootstrap replicates 1000 was set to obtain a reliable tree. The Galaxy server-based IQTREE program was used to infer the phylogeny relatedness (Minh et al., 2020). The treefile format from IQTREE was visualized by using Interactive Tree of Life (iTOL). The tree was rooted with mid-point rooting, and tree branches were annotated with host, isolation source, country and year of isolation, respectively (Letunic and Bork, 2021).

### 2.4 Pangenome analysis of *M. orygis*

The pan genomic analysis of *M. orygis* (n= 53) was carried out with Roary and Panaroo pan genome pipeline (Page et al., 2015). *M. orygis* strain 115(1)C was not used for the pangenome analysis due to poor quality of genome. Genome assemblies of *M. orygis* were annotated with Prokka. The GFF outputs from Prokka were used for Roary and Panaroo pan genomic pipeline. Pangenomic tools screen and sort out every isolate based on gene presence or absence (Page et al., 2015; Tonkin-Hill et al., 2020). The CSV output from Roary and Panaroo was used to visualize the pangenome with FriPan (http://drpowell.github.io/FriPan/), and open and closeness of the genome were calculated using PanGP. Fripan is an interactive web tool to analyze and group bacterial strains based on the accessory genomes. Script roary2fripan (https://github.com/kwongj/roary2fripan) was used to convert the CSV file of Roary to FriPan formats. Rtab binary file from Roary was used for the PanGP analysis. The complete pan-genome profile analysis was carried out using the Heaps law (y = A_pan_ x B_pan_ +C_pan_) formula. The PanGP tool works based on the DG algorithm to calculate the genome diversity of the population (Zhao et al., 2014). The results of Roary and Panaroo were compared to indicate the effectiveness of pipelines. Core genome phylogeny was constructed using the Panaroo generated core genome alignment. Panaroo identified and aligned the conserved orthologous genes from available genomes. A further maximum likelihood tree was constructed with 1000 bootstraps by using the iqtree webserver. Interactive web tool iTol was used for the visualization of core genome phylogeny.

### 2.5 Genotyping

Spoligotyping is a spacer oligonucleotide typing which is widely used for genotyping *Mycobacterium* spp. Lorikeet is the tool for digital spoligotyping of mycobacterial genomes from Illumina reads. The WGS raw reads from *M. orygis* strains (n=53) were subjected to lorikeet spoligotyping to determine the lineage of strains (Cohen et al., 2015). Multilocus sequence typing is sequence-based genotyping, utilizing multiple housekeeping genes to classify the bacterial strains. Sequence types and allelic profiles of *M. orygis* genomes were identified using galaxy server-based MLST (version 2.22.0) tool. Available mycobacteria pubMLST schemes with a minimum DNA identity of 95% and DNA coverage of 10% were used for determining similar allelic profiles and sequence typing (Seemann, 2016).

### 2.6 Region of difference analysis

MTBC species are classified based on the SNPs and large polymorphisms present in the region of difference (RD). Presence or absence of RD in the NGS sequence reads can be used to predict the lineage of strains (Bespiatykh et al., 2021). RDscan (https://github.com/dbespiatykh/RDscan) was used to determine the deletion regions (RDs) in all the *M. orygis* used in the study (n= 55). Sequence reads of the genomes were extracted and downloaded through the Galaxy Europe server. RDscan script was executed with snakemake python script in Ubuntu 20.04.

### 2.7 Single nucleotide polymorphism analysis

The *M. orygis* genomes from NCBI and our study were subjected to SNP analysis. Sequence reads were directly extracted from the NCBI to the galaxy server. Sequence reads were trimmed and quality was assessed with trimmometric and FastQC tools. Mapping and variant calling was performed with Snippy 4.6.0 software. The software uses BWA for read mapping and variant calling with the *M. tuberculosis* (H37Rv) reference genome (Seemann, 2020). The VCF file obtained from Snippy was merged and filtered with the TB variant filter tool with region, snv, and depth. The TB variant report was also prepared with Galaxy server tools and analyzed for SNPs and indels. The genes predicted with SNPs were evaluated for the ortholog analysis using the EggNOG webserver. Fasta sequences of SNP-containing genes were extracted and classified for the subsystems (Huerta-Cepas et al., 2016).

## 3 Results

**Figure 1:**
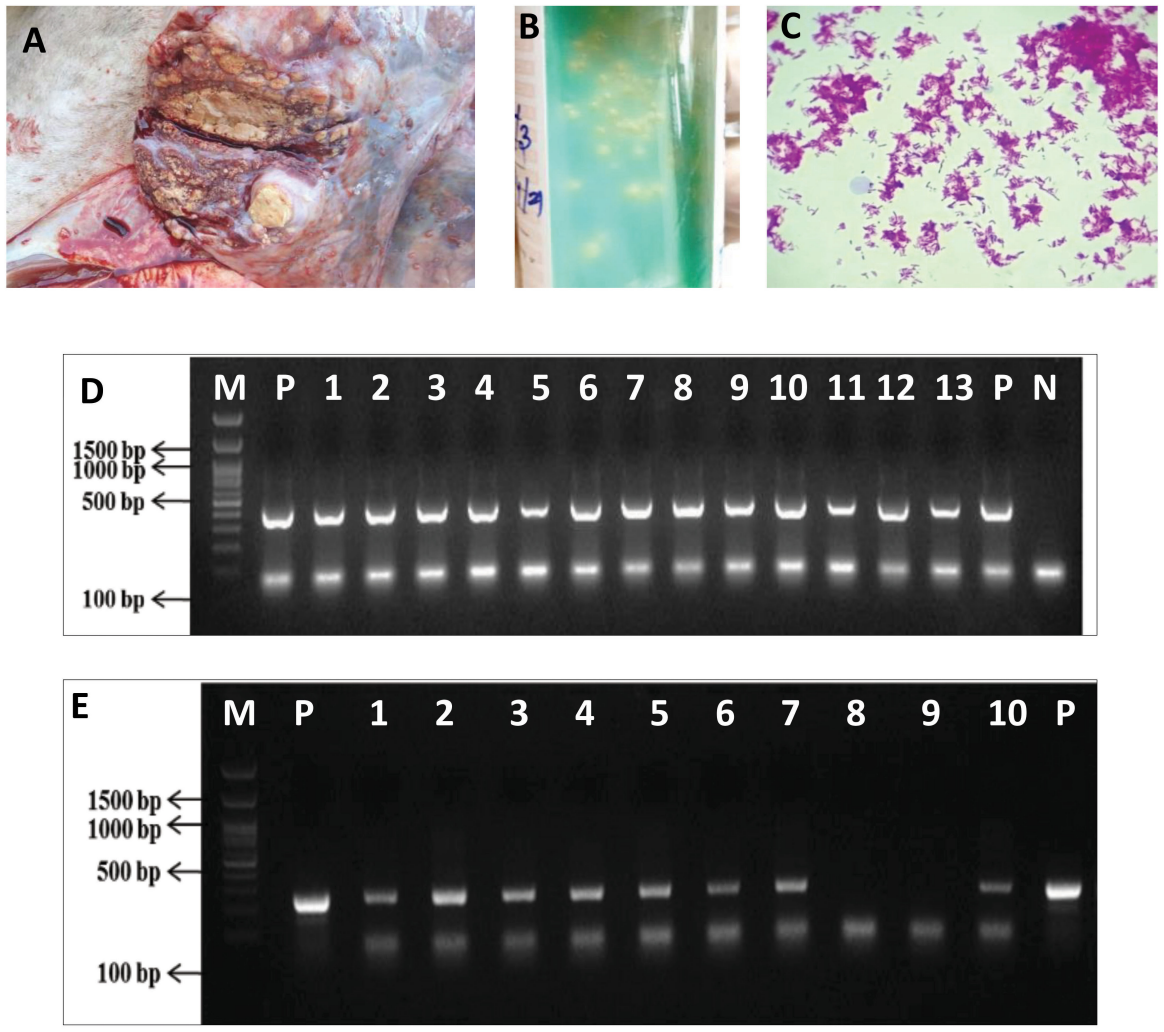
Isolation and confirmation of *M. orygis* from clinical samples. **(A)** Caseous nodule in the lung of cattle (ID Jx25). **(B)** *Mycobacterium* colonies on LJ slant. **(C)** Acid-fast positive bacilli from LJ slant. **(D)** IS6110 PCR for confirmation of MTBC. Lane P—Positive control (*M. bovis* AN5), Lane N—Negative control, Lanes 1 to 9—*M. orygis* TANUVAS_1, TANUVAS_2, and TANUVAS_4 to TANUVAS_10. Lanes 10 and 11—*M. bovis* BCG strain. Lanes 12 and 13—*M. tuberculosis*. **(E)** RD12 PCR for confirmation of *M. orygis.* Lane P—positive control (*M. orygis*). Lanes 1 to 7—*M. orygis* TANUVAS_1, TANUVAS_2, and TANUVAS_4 to TANUVAS_8. Lane 8—*M. tuberculosis*. Lane 9—*M. bovis* BCG. Lane 10—*M. orygis* TANUVAS_9.

A total of nine MGIT cultures showed acid-fast positive bacilli, and *Mycobacterium*-specific colonies were observed on LJ slant. All the nine isolates showed 445 bp product size for IS*6100* PCR, depicting the isolates as MTBC. RD12 region-based PCR showed amplification of 264 bp confirming the isolates as *M. orygis* (**Figure 1**). *M. orygis*-positive isolates were recovered from samples of four cattle, four deer, and one buffalo. The features of all the nine whole genomes carried out in this study are provided in **Supplementary Table 2**.

**Figure 2:**
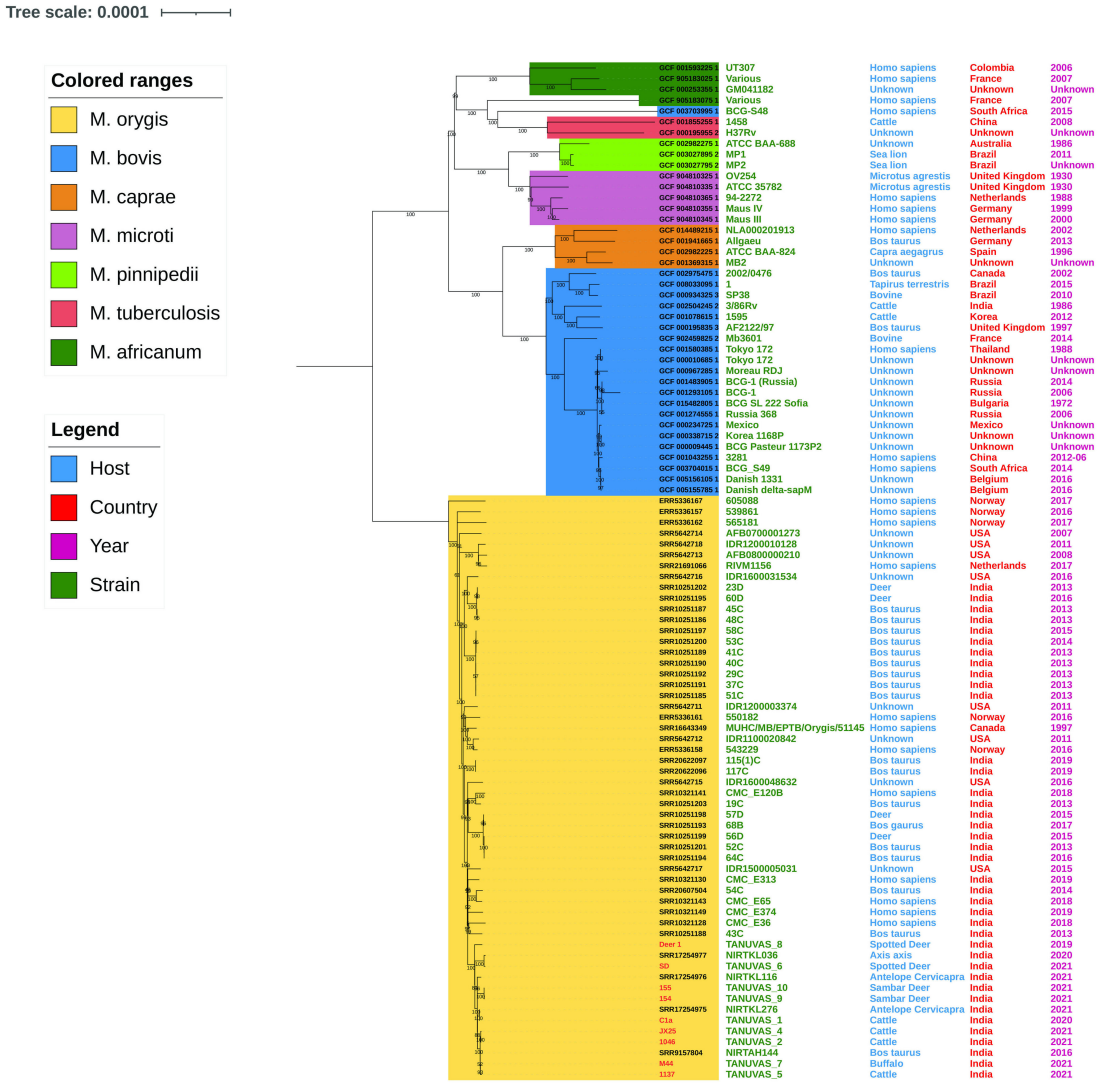
Whole genome phylogeny of MTBC strains (n= 94). Colored ranges indicate different MTBC members, and host, country, and year of isolation are marked adjacent to the strains. *M. orygis* forms a separate cluster from the rest of the MTBC members.

All *M. orygis* strains used in the analysis clearly separated from the other members of the *Mycobacterium tuberculosis* complex (MTBC) based on whole genome phylogeny (**Figure 2**). All *M. orygis* strains recovered in the study were clustered together. Strains TANUVAS_2, TANUVAS_4, TANUVAS_5, TANUVAS_7, and NIRTAH144 were genetically more closely related. Similarly, TANUVAS_9 and TANUVAS_10 were more closely related, while TANUVAS_6 was related closely to NIRTKL036, and TANUVAS_1 was related to NIRTKL276. There was no geographical distinction or host-based distinction based on whole genome phylogeny.

In the present study, Pairwise SNP were below 100 when compared between strains isolated from Tamil Nadu, India (present study or earlier report). Comparison of strains from other parts of India with Tamil Nadu strains showed SNPs between 100 and 200. Similarly, strains from Norway, USA, and the Netherlands showed higher SNPs (120–1064) when compared with Indian strains (**Supplementary Table 3**). A maximum of 1064 SNPs was found between strains 565181 and TANUVAS_4. Strains TANUVAS_2, TANUVAS_4, TANUVAS_5, TANUVAS_7, and NIRTAH144 had SNPs less than 10, while other strains from this study had SNPs more than 25. Among the 10 *M. orygis* strains isolated in the study, all strains except TANUVAS_6 were predicted with 66 virulence genes. Gene *mgtC* was not predicted in TANUVAS_6, while the *clpP* gene was not predicted in any the 10 *M. orygis* strains.

### 3.1 Typing

Based on MLST, all the *M. orygis* strains used in the study belonged to ST215 except strain IDR1600048632, which was untyped. Spoligotyping using SpolPred tool showed that all the *M. orygis* strains had a similar binary pattern (1100000001111000000000001111111101001101111), except strains MUHC/MB/EPTB/Orygis/51145 (1100000000000000000000000000000000000101111), 565181 (1000000001110000000000001111111111001101111), and 43C (1100000001111000000000000110000000000001111) (**Supplementary Table 4**). There was no spoligotype international type (SIT) assigned for the binary pattern. On further manual checking of the spacer regions, spacer 2 and 3 was found in all *M. orygis*, while spacer 33 was absent in MUHC/MB/EPTB/Orygis/51145, 52C, 68B, 64C, and 43C. Hence, all the *M. orygis* strains except MUHC/MB/EPTB/Orygis/51145, 52C, 68B, 64C, and 43C were assigned with SIT 587.

### 3.2 Pangenome analysis of *M. orygis*

**Figure 3:**
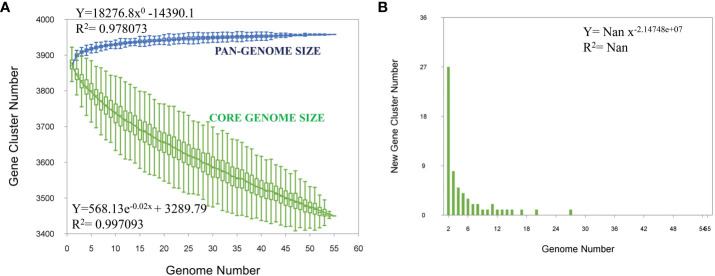
Pangenome analysis. **(A)** Core and pangenome analysis for 53 *M. orygis* strains analyzed. Total number of core (green) and pan (blue) genome are plotted. The pangenome curve has attained a plateau, indicating that *M. orygis* has a closed pangenome structure. **(B)** Strain specific gene analysis for *M. orygis* strains. Number of new genes added to the gene pool on addition of new genome is plotted.

Pangenome analysis of the 53 *M. orygis* strains showed a total of 3976 genes, 3031 core genes, 724 soft core genes, 156 shell genes, and 65 cloud genes (accessory genome). The *Bpan* value of the pan genome is 0 showing that it is a “closed” pangenome. The pangenome curve shows a plateau indicating the “closed” pangenome structure. There was no addition of new genes upon addition of new genome to the pangenome analysis (**Figure 3**). There was no clear discrimination of strains based on geographical location or year of isolation (**Supplementary Figure 1**). Clustering of *M. orygis* in core genome phylogeny was similar to whole genome phylogeny (**Supplementary Figure 2**).

### 3.3 SNP analysis

A total of 8696 SNPs were predicted when the *M. orygis* genome was compared with the reference genome *M. tuberculosis* H37Rv. In total, 66 SNPs were predicted to affect the genes heavily. These SNPs belong to start lost, stop gained, stop lost, and stop lost; splice region variant. Multiple intragenic variant SNPs were observed in the tRNA(ile) region. A total of 1700 non-synonymous SNPs were predicted and were used for identifying the COG functional classification. Several genes (n= 439) were not predicted with functional category; 105 genes belonged to the energy production and conversion categories (C); 115 genes belonged to lipid metabolism (I); 92 genes belonged to amino acid metabolism and transport (E); 98 genes belonged to replication, recombination, and repair (L); 46 genes belonged to secretion, motility, and chemotaxis (N); 73 genes belonged to inorganic ion transport and metabolism (P); 86 genes belonged to secondary metabolites biosynthesis, transport, and catabolism (Q); 13 genes belonged to intracellular trafficking, secretion, and vesicular transport (U); and 34 genes belonged to defense mechanism (V) (**Supplementary Table 5**).

The SNPs present in the virulence factors were analyzed, and the majority of the genes were shown to share the synonymous and non-synonymous mutations. A total of 378 SNPs were present in the 165 genes, among which 238 missense and 138 synonymous SNPs were recorded. Secretion system (PPE4, PE35) and cell surface components (*fad*13, MRA2983) genes gained stop codons, whereas *mps*1 lost the stop codon. All the *M. orygis* strains analyzed gained a stop codon in the PE35 gene before the actual stop codon (**Supplementary Figure 3**). Initiator codon variant was noticed in the copper uptake gene (*ctp*V). The genes categorized as regulation shared non-synonymous SNPs. These non-synonymous SNP and stop codons may cause a high or moderate impact on the expression of virulence factor-associated genes (**Supplementary Table 6**).

### 3.4 Region of difference

**Figure 4:**
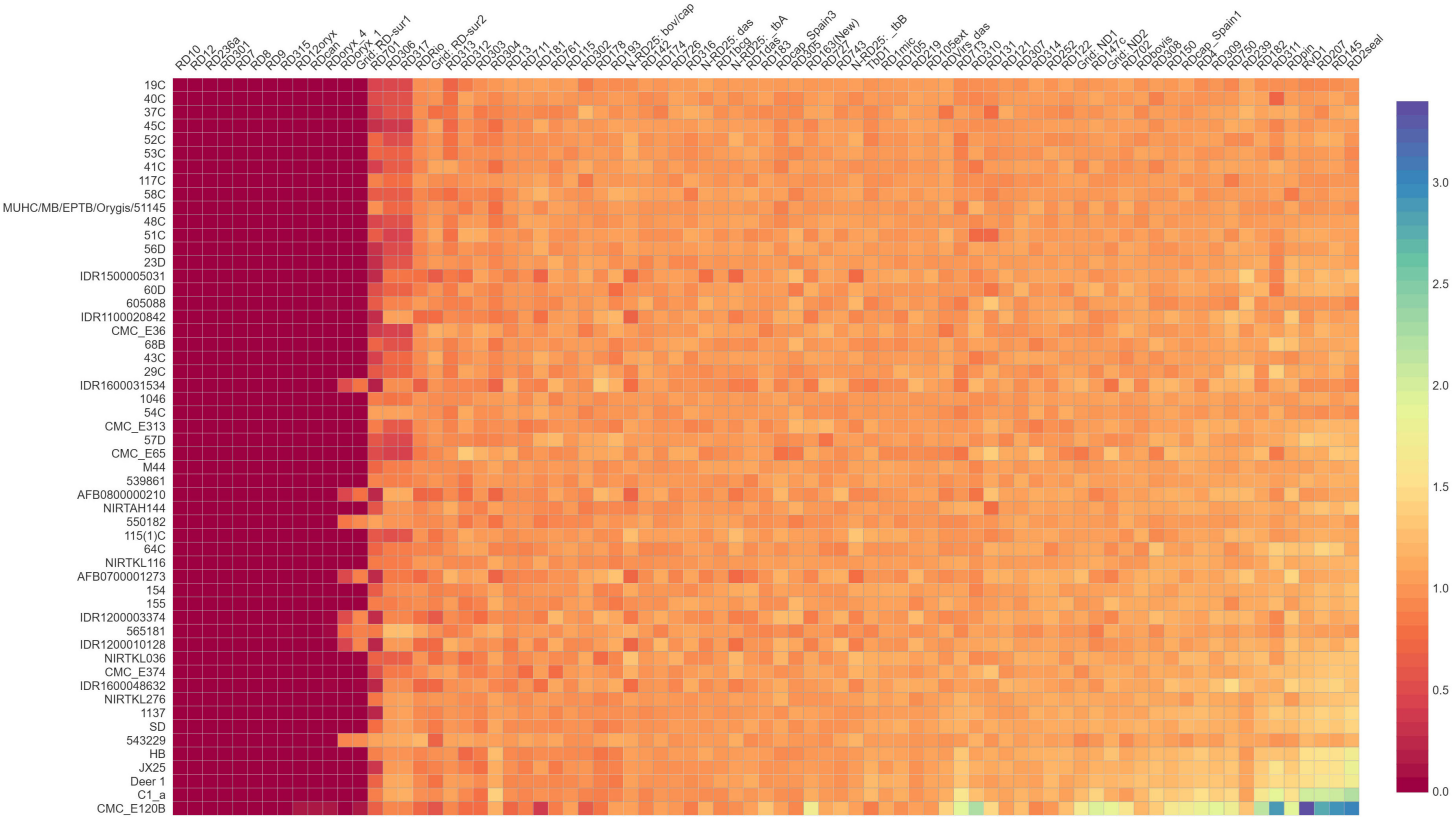
Region of difference among *M. orygis* strains. Values obtained from RDscan are plotted in a heat map depiction. Dark violet color indicates absence of the region (RDscan value= 0), while orange/yellow color indicates presence of the region (RDscan value >0.5 up to 3). RD10, RD12, RD236a, RD7, RD8, RD12oryx, RD301, and RD315 are absent in all the *M. orygis* genome analyzed.

A total of 79 regions were predicted by RDscan in all the *M. orygis* strains, except RIVM1156 where there were no RDs predicted. RD7, RD8, RD9, RD10, RD12, RD236a, RD301, RD315 were not present in any of the *M. orygis* (n= 53) analyzed. RD12oryx, RDcan, and RDoryx_4 were not predicted in all *M. orygis* strains except CMC_E120B. RDOryx_1 and RD-Sur1 were predicted only in IDR1200010128, AFB0800000210, 550182, AFB0700001273, IDR1200003374, 565181, IDR1200010128, and 543229 (**Figure 4**).

## 4 Discussion

Studies from India have shown the presence of *M. orygis* in humans (Duffy et al., 2020) and animal species such as cattle, spotted deer, black buck, and Indian bison (Refaya et al., 2022; Sharma et al., 2023), suggesting that there is need for a nationwide surveillance of this pathogen to elucidate its role in tuberculosis. Isolation of *M. orygis* but not *M. bovis* in this study correlates with earlier reports from India. To the best of our knowledge, this report on the isolation and whole genome sequencing of *M. orygis* from water buffalo (*Bubalus bubalis*) and sambar deer (*Cervus unicolor*) is the first of its kind.

The whole genome phylogeny shows three major clades of *Mycobacterium* spp. affecting animals: *M. orygis* forming a separate clade (I), *M. bovis* and *M. caprae* together forming the second clade, and *M. microti* and *M. pinnipedii* forming the third clade, as reported by Brites et al. (2018). Strains TANUVAS_2, TANUVAS_4, TANUVAS_5, TANUVAS_7, and NIRTAH144 were isolated from same geographical location, which may be the reason for genetic relatedness. There may be possibility that same *M. orygis* strain might be the reason for disease in the same geographical location (Tamil Nadu) since SNP cut-off for disease transmission among the affected animals was 3–14 (Walker et al., 2013). Similarly, 0–6 SNPs between *M. orygis* strains were reported earlier in human patients, suggesting the possible role of single source infection (Lipworth et al., 2019).

Pangenome analysis for *M. orygis* has not been reported previously, and hence it has been attempted in this study. The pangenome of *M. orygis* was found to be closed, which was similar to the closed nature of *M. bovis* reported previously (Ceres et al., 2022). The genome structure of *M. orygis* is compact, and genes that are essential for survival are present. Since a fewer number of accessory genomes are present, the chances for genetic exchange are lower. Pangenome analysis of *M. orygis* using the Roary pan-genome pipeline showed an open pangenome structure (date not shown), while the Panaroo pipeline showed a closed structure. Similarly, Reis and Cunha (2021) reported an open pangenome structure of *M. bovis* using Roary, while Ceres et al. (2022) reported closed a pangenome by using Panaroo. Hence, in the present study, the results obtained from Panaroo were used.

Similar to this study, *M. orygis* was assigned with spoligotype SIT587, while SIT701 and orphan types were also reported (Gey van Pittius et al., 2012; Riojas et al., 2018). Since only nine *M. orygis* strains were available, new spoligotype patterns predicted for strains like MUHC/MB/EPTB/Orygis/51145 using bioinformatics could not be confirmed using hybridization assay, which is a limitation of the study. Subsequently, *Mycobacterium* spp. has less genetic diversity, and MLST and spoligotyping may not be useful for genotyping or construction of phylogeny; instead, large sequence polymorphisms (LSPs) or RDs can be used (Bespiatykh et al., 2021). RD301 and RD315, which were reported to be uniquely absent in *M. orygis* (Bespiatykh et al., 2021), were absent in all the *M. orygis* genomes tested in the study. Contradictory to earlier findings (Bespiatykh et al., 2021; Liu Z. et al., 2022), RD236a was absent in all the *M. orygis* genome analyzed. As reported earlier, RD8 (comprising of Rv3617, Rv3618, Rv3619c, Rv3620c, Rv3621c, Rv3622c, and Rv3623 affected genes) is absent in all *M. orygis*, and RD236a corresponds to the Rv3617 (*ephA*) gene. Since the Rv3617 gene is common in both RDs, RD236a might not have been predicted in the *M. orygis* genome. Blast analysis of the *M. orygis* genome used in this study also showed the absence of Rv3617.

Prediction of RDoryx_1 in 7/32 *M. orygis* at other regions of the genome was reported earlier (Bespiatykh et al., 2021), and that may be the reason for the prediction of RDoryx_1 in eight strains in this study. RDoryx_1 comprises eight gene deletions, while RD-Sur1 has 15 gene deletions that includes the eight genes of RDoryx_1 (Liu Z. et al., 2022). Since the genes for RDoryx_1 were predicted in eight strains and similar genes correspond to the RDSur-1 region, RDSur-1 might have been predicted in these eight strains. Bespiatykh et al. (2021) analyzed 32 *M. orygis* strains, while in the present study, 54 strains were used for RD analysis. Based on both the studies, RD301 and RD315 were specifically absent in all the *M. orygis* analyzed. The same was confirmed by amplifying the RD301 region using 14 *M. orygis* genomes (nine isolated in this study and five isolates available in the laboratory), which showed no amplification by PCR. The same region had specific PCR amplification for *M. tuberculosis* and *M. bovis* BCG (**Supplementary Figure 4**). There are reports regarding the misidentification of *M. orygis* as *M. africanum* since better diagnostic tools are not available to differentiate the MTBC. Since MTBC members are more closely related, a better diagnostic tool is essential to identify *M. orygis* (Rahim et al., 2007; Islam et al., 2023). Hence, RD301 or RD315 can be used as a marker for identifying or discriminating *M. orygis* from other members of MTBC. Since only a limited number of *M. orygis* genomes were available while drafting the manuscript, analysis with additional datasets from different geographical regions would help to improve confidence regarding the RDs.

A higher number of synonymous SNPs was identified in the *M. orygis*. Non-synonymous SNPs were identified in the genes related to the lipid and energy metabolism. Similar types of non-synonymous SNPs were detected in the *M. bovis* strains originating from Spain and France (Hauer et al., 2019; Perea et al., 2021). Non-synonymous SNPs may affect the structure and arrangement of lipid and protein virulence factors. SNPs in these genes may be involved in the evolution, persistence, and transmission of *M. orygis* in wild and domestic animals of tropical countries including India. Further, the impact of SNPs on the phenotypic nature of *M. orygis* needs to be studied to understand the pathogenesis of this bacterium in humans and animals.

The genes PPE4 and PE35 are linked with the ESX1 and ESX3 secretion system of mycobacterial species. These genes gained a stop codon in *M. orygis*, which will alter the amino acid sequences of the protein. PE/PPE gene products were related to bacterial virulence and involved in host–pathogen interactions such as invasion, immune regulation, and intracellular survival (Qian et al., 2020). PPE4 carry out iron or zinc acquisition from the cells and regulate mycobactin utilization (Tufariello et al., 2016). PE35 is a proline-rich protein and is encoded in RD1 region. PE35 is important for *M. tuberculosis* infection and cellular-level response. Due to the partial loss of RD1 in the BCG strain, it is used to differentiate vaccinated and infected humans (Jiang et al., 2016). Polymorphism in this gene region affects intracellular survival and immune regulation during infection and could be employed as a marker in future diagnostic tools.

## 5 Conclusion

This study supports that hypothesis that *M. orygis* is the most prevalent *Mycobacterium* spp. in animals in India. The study is also the first to report *M. orygis* in water buffalo and sambar deer. Compared to spoligotyping and MLST, RD-based analysis discriminates *M. orygis* from other MTBC members, and RD301 and RD315 as validated by PCR can be used as diagnostic markers for confirmation of the presence of *M. orygis*. Similarly, SNPs in the PE35 gene can be used as molecular markers for the identification of *M. orygis*.

## Data availability statement

All the genomes generated in this study have been submitted tothe NCBI SRA database with the bio project number PRJNA785380.

## Ethics statement

The study does not involve animal trials, and samples receivedat the laboratory were used for isolation, hence, this study does notrequire approval for conducting animal trials.

## Author contributions

KK: Conceptualization, Formal analysis, Investigation, Methodology, Writing – original draft, Writing – review & editing. SS: Data curation, Investigation, Methodology, Writing – review & editing. MV: Data curation, Investigation, Methodology, Writing – review & editing. AJ: Data curation, Methodology, Writing – review & editing. SA: Methodology, Software, Writing – original draft, Writing – review & editing. RK: Methodology, Resources, Writing – review & editing. PT: Software, Validation, Writing – review & editing. RA: Data curation, Supervision, Writing – review & editing. KGT: Funding acquisition, Resources, Validation, Writing – review & editing. GD: Funding acquisition, Project administration, Resources, Writing – review & editing.

## Supplementary material

Supplementary Table 3 Pairwise SNP distance of *M. orygis* genomes used in the study.

Supplementary Table 4 Spoliogotyping results of the *M. orygis* genomes.

Supplementary Table 5 COG categorization of functional genes that possess missense mutation.

Supplementary Figure 1 Pangenome-based phylogeny and multidimensional scaling. A. Pangenome-based phylogeny. B. Multidimensional scaling of *M. orygis* genome. No major categorization based on place, year, or host of isolation.

Supplementary Figure 2 Core genome phylogeny of MTBC isolates used in this study.

Supplementary Figure 3 Amino acid sequence alignment of PE35 gene showing stop codon (gained) marked as *. The first sequence is the *M. tuberculosis* reference sequence to which all the *M. orygis* is compared.

Supplementary Figure 4 Representative gel electrophoresis image of PCR for RD307 and RD315. A. *M. tuberculosis* DNA. Lane 1—RD301, Lane 2—RD315. B. *M. bovis* BCG and *M. orygis* DNA. Lanes 1 and 2—*M. bovis* BCG and *M. orygis* tested for RD301 region respectively. Lanes 3 and 4—*M. bovis* BCG and *M. orygis* tested for RD315 region respectively.
