# Supplementary material for: Whole genome sequencing and comparative genomics of Mycobacterium orygis isolated from different animal hosts to identify specific diagnostic markers
Source: Front Cell Infect Microbiol. 2023 Dec 22;13:1302393. doi: 10.3389/fcimb.2023.1302393 (PMC10770871; doi:10.3389/fcimb.2023.1302393)
Supplement: Supplementary file 2 [file DataSheet_2.docx]

| **Sample ID.** | **Strain ID** | **Contigs** | **Bases** | **CDS** |
| --- | --- | --- | --- | --- |
| C1 | TANUVAS_1 | 116 | 4279443 | 3901 |
| 1046 | TANUVAS_2 | 129 | 4255414 | 3864 |
| JX25 | TANUVAS_4 | 109 | 4245631 | 3885 |
| 1137 | TANUVAS_5 | 108 | 4238423 | 3894 |
| Deer 5 | TANUVAS_6 | 91 | 4283678 | 3896 |
| M44 | TANUVAS_7 | 91 | 4283909 | 3888 |
| Deer 1 | TANUVAS_8 | 98 | 4284757 | 3898 |
| Deer 3 | TANUVAS_9 | 92 | 4281297 | 3894 |
| Deer 4 | TANUVAS_10 | 117 | 4215883 | 3831 |

**Supplementary Table 2. Whole genome features of *M. orygis* strains isolated in this study**
