# Supplementary material for: Whole genome sequencing and comparative genomics of Mycobacterium orygis isolated from different animal hosts to identify specific diagnostic markers
Source: Front Cell Infect Microbiol. 2023 Dec 22;13:1302393. doi: 10.3389/fcimb.2023.1302393 (PMC10770871; doi:10.3389/fcimb.2023.1302393)
Supplement: Supplementary file 6 [file DataSheet_6.docx]

**Supplementary Table 6. SNPs in virulence genes of *M. orygis*.** Synonymous, nonsynonymous and stop gained or lost SNPs are listed corresponding to the virulence mechanism. ✔ mark indicates presence of the SNP in the gene.

| **Group** | **Product** | **Gene** | **Locus Tag** | **Syn-SNP** | **Miss-SNP** | **STOP_GAIN/LOST** |
| --- | --- | --- | --- | --- | --- | --- |
| **Amino acid and purine metabolism** | Glutamine synthesis | *glnA1* | MRA_2237 |  | ✔ |  |
|  | Lysine synthesis | *lysA* | MRA_1301 | ✔ |  |  |
|  | Proline synthesis | *proC* | MRA_0507 | ✔ | ✔ |  |
| **Anaerobic respiration** | Fused nitrate reductase | *narX* | MRA_1747 |  | ✔ |  |
|  | Nitrate reductase | *narG* | MRA_1172 | ✔ | ✔ |  |
|  |  | *narH* | MRA_1173 | ✔ | ✔ |  |
|  | Nitrate/nitrite transporter | *narK2* | MRA_1748 |  | ✔ |  |
| **Anti-apoptosis factor** | NuoG | *nuoG* | MRA_3184 |  | ✔ |  |
| **Catabolism of cholesterol** | Cyp125 | *cyp125* | MRA_3584 | ✔ |  |  |
| **Cell surface components** | Carboxylesterase | *caeA* | MRA_2241 | ✔ |  |  |
|  |  | *fad23* | MRA_1195 |  | ✔ |  |
|  |  | *fadE5* | MRA_0253 | ✔ |  |  |
|  |  | *gtf1* | MRA_1538 | ✔ |  |  |
|  |  | *gtf2* | MRA_1536 | ✔ | ✔ |  |
|  |  | *mmpL10* | MRA_1193 | ✔ | ✔ |  |
|  |  | *mmpS4* | MRA_0456 | ✔ |  |  |
|  |  | *mps1* | MRA_0106 | ✔ | ✔ | STOP_LOST |
|  |  | *papA3* | MRA_1192 | ✔ | ✔ |  |
|  |  | *pe* | MRA_1194 | ✔ | ✔ |  |
|  | Heparin-binding hemagglutinin | *hbhA* | MRA_0482 |  | ✔ |  |
|  | Methyltransferase | *mmaA4* | MRA_0653 |  | ✔ |  |
|  | Mycolic acid trans-cyclopropane synthetase | *cmaA2* | MRA_0511 |  | ✔ |  |
|  | MymA operon | *adhD* | MRA_3118 | ✔ | ✔ |  |
|  |  | *chp* | MRA_3119 |  | ✔ |  |
|  |  | *fadD13* | MRA_3121 | ✔ |  | STOP_GAIN |
|  |  | *lipR* | MRA_3116 | ✔ | ✔ |  |
|  |  | *mymA* | MRA_3115 |  | ✔ |  |
|  |  | *tgs4* | MRA_3120 | ✔ |  |  |
|  |  | *Undetermined* | MRA_2985 | ✔ |  |  |
|  |  | *Undetermined* | MRA_2989 | ✔ |  |  |
|  |  | *Undetermined* | MRA_2976 | ✔ |  |  |
|  |  | *Undetermined* | MRA_2982 | ✔ |  |  |
|  |  | *Undetermined* | MRA_2983 |  | ✔ | STOP_GAIN |
|  |  | *ddrA* | MRA_2962 |  | ✔ |  |
|  |  | *fadD22* | MRA_2975 | ✔ | ✔ |  |
|  |  | *fadD26* | MRA_2956 |  | ✔ |  |
|  |  | *fadD28* | MRA_2967 | ✔ |  |  |
|  |  | *fadD29* | MRA_2977 | ✔ | ✔ |  |
|  |  | *lppx* | MRA_2972 |  | ✔ |  |
|  |  | *mas* | MRA_2966 | ✔ | ✔ |  |
|  |  | *mmpL7* | MRA_2968 | ✔ | ✔ |  |
|  |  | *pks1* | MRA_2973 |  | ✔ |  |
|  |  | *ppsA* | MRA_2957 | ✔ | ✔ |  |
|  |  | *ppsB* | MRA_2958 | ✔ | ✔ |  |
|  |  | *ppsC* | MRA_2959 | ✔ | ✔ |  |
|  |  | *ppsD* | MRA_2960 | ✔ | ✔ |  |
|  |  | *ppsE* | MRA_2961 | ✔ | ✔ |  |
|  |  | *tesA* | MRA_2955 |  | ✔ |  |
|  | Potassium/proton antiporter | *kefB* | MRA_3277 | ✔ |  |  |
|  | Proximal cyclopropane synthase of alpha mycolates | *pcaA* | MRA_0476 | ✔ |  |  |
|  | Sulfolipid-1 biosynthesis and transport | *mmpL8* | MRA_3863 | ✔ | ✔ |  |
|  |  | *papA1* | MRA_3864 | ✔ | ✔ |  |
|  |  | *papA2* | MRA_3860 |  | ✔ |  |
|  |  | *pks2* | MRA_3865 | ✔ | ✔ |  |
|  |  | *stf0* | MRA_0304 | ✔ |  |  |
|  | Trehalose-recycling ABC transporter | *chp1* | MRA_3862 | ✔ | ✔ |  |
|  |  | *fad23* | MRA_3866 | ✔ | ✔ |  |
|  |  | *lpqY* | MRA_1244 | ✔ |  |  |
|  |  | *icl2* |  |  | ✔ |  |
|  |  | *sugB* | MRA_1246 |  | ✔ |  |
| **Copper uptake** | Copper exporter | *ctpV* | MRA_0976 | ✔ | ✔ | initiator_codon_variant |
| **Iron uptake** | ABC transporter | *irtA* | MRA_1356 | ✔ | ✔ |  |
|  |  | *irtB* | MRA_1357 | ✔ | ✔ |  |
|  | Heme uptake | *Undetermined* | MRA_0211 | ✔ |  |  |
|  |  | *mmpL11* | MRA_0210 | ✔ |  |  |
|  |  | *mmpL3* | MRA_0214 | ✔ | ✔ |  |
|  | Iron-dependent regulator | *ideR* | MRA_2739 |  | ✔ |  |
|  |  | *fadD33* | MRA_1353 |  | ✔ |  |
|  |  | *mbtB* | MRA_2406 | ✔ | ✔ |  |
|  |  | *mbtC* | MRA_2405 | ✔ |  |  |
|  |  | *mbtD* | MRA_2404 |  | ✔ |  |
|  |  | *mbtE* | MRA_2403 |  | ✔ |  |
|  |  | *mbtF* | MRA_2402 | ✔ | ✔ |  |
|  |  | *mbtG* | MRA_2401 |  | ✔ |  |
|  |  | *mbtH* | MRA_2400 |  | ✔ |  |
|  |  | *mbtI* | MRA_2409 | ✔ |  |  |
| **Lipid and fatty acid metabolism** | Isocitrate lyase | *icl* | MRA_0473 | ✔ |  |  |
|  | Lipase | *lipF* | MRA_3527 |  | ✔ |  |
|  | Lipid phosphatase | *sapM* | MRA_3351 |  | ✔ |  |
|  |  | *panD* | MRA_3640 |  | ✔ |  |
|  |  | *plcB* | MRA_2370 | ✔ |  |  |
|  |  | *plcC* | MRA_2369 | ✔ |  |  |
| **Magnesium uptake** | Magnesium transport | *mgtC* | MRA_1823 | ✔ | ✔ |  |
| **Mammalian cell entry (mce) operons** | Mce1 | *mce1A* | MRA_0177 | ✔ | ✔ |  |
|  |  | *mce1B* | MRA_0178 |  | ✔ |  |
|  |  | *mce1C* | MRA_0179 | ✔ | ✔ |  |
|  |  | *mce1D* | MRA_0180 | ✔ |  |  |
|  |  | *mce1E* | MRA_0181 | ✔ | ✔ |  |
|  |  | *mce1F* | MRA_0182 | ✔ | ✔ |  |
|  | Mce2 | *mce2A* | MRA_0596 |  | ✔ |  |
|  |  | *mce2B* | MRA_0597 | ✔ |  |  |
|  |  | *mce2C* | MRA_0599 | ✔ | ✔ |  |
|  |  | *mce2F* | MRA_0602 | ✔ | ✔ |  |
|  | Mce4 | *mce4C* | MRA_3537 | ✔ |  |  |
|  |  | *mce4E* | MRA_3535 | ✔ | ✔ |  |
|  |  | *mce4F* | MRA_3534 |  | ✔ |  |
| **Manganese uptake** | P(1)-type Mn2+ transporting ATPase | *ctpC* | MRA_3311 | ✔ | ✔ |  |
| **Phagosome arresting** | PE family protein | *PE_PGRS30* | MRA_1662 | ✔ | ✔ |  |
| **Protesase** | Zn++ metallophrotease | *zmp1* | MRA_0206 | ✔ |  |  |
|  | DevR/S | *devS* | MRA_3164 |  | ✔ |  |
|  | MprA/B | *mprA* | MRA_0988 |  | ✔ |  |
|  |  | *mprB* | MRA_0989 |  | ✔ |  |
|  | PhoP/R | *phoP* | MRA_0767 |  | ✔ |  |
|  |  | *phoR* | MRA_0768 |  | ✔ |  |
|  | PrrA/B | *prrA* | MRA_0910 |  | ✔ |  |
|  |  | *prrB* | MRA_0909 |  | ✔ |  |
|  | Sigma H | *sigH* | MRA_3263 |  | ✔ |  |
|  | Sigma L | *sigL* | MRA_0743 |  | ✔ |  |
|  | Sigma M | *sigM* | MRA_3950 |  | ✔ |  |
| **Secreted proteins** | Antigen 85 complex | *fbpA* | MRA_3844 |  | ✔ |  |
|  |  | *fbpB* | MRA_1897 |  | ✔ |  |
|  | Protein kinase G | *pknG* | MRA_0416 | ✔ | ✔ |  |
| **Secretion system** | Accessory secretion factor | *secA2* | MRA_1833 |  | ✔ |  |
|  | ESX-1 (T7SS) | *PE35* | MRA_3911 | ✔ | ✔ | STOP_GAIN |
|  |  | *PPE68* | MRA_3912 |  | ✔ |  |
|  |  | *eccA1* | MRA_3907 | ✔ |  |  |
|  |  | *eccB1* | MRA_3908 | ✔ | ✔ |  |
|  |  | *eccCa1* | MRA_3909 | ✔ | ✔ |  |
|  |  | *eccCb1* | MRA_3910 | ✔ | ✔ |  |
|  |  | *eccD1* | MRA_3916 | ✔ |  |  |
|  |  | *eccE1* | MRA_3921 |  | ✔ |  |
|  |  | *espA* | MRA_3652 | ✔ | ✔ |  |
|  |  | *espB* | MRA_3920 | ✔ | ✔ |  |
|  |  | *espC* | MRA_3651 |  | ✔ |  |
|  |  | *espD* | MRA_3650 | ✔ |  |  |
|  |  | *espE* | MRA_3903 |  | ✔ |  |
|  |  | *espF* | MRA_3904 |  | ✔ |  |
|  |  | *espI* | MRA_3915 |  | ✔ |  |
|  |  | *espJ* | MRA_3917 | ✔ | ✔ |  |
|  |  | *espK* | MRA_3918 |  | ✔ |  |
|  | ESX-2 (T7SS) | Undetermined | MRA_3927 | ✔ | ✔ |  |
|  |  | *PE36* | MRA_3932 |  | ✔ |  |
|  |  | *PPE69* | MRA_3931 |  | ✔ |  |
|  |  | *eccA2* | MRA_3923 | ✔ | ✔ |  |
|  |  | *eccB2* | MRA_3934 | ✔ | ✔ |  |
|  |  | *eccC2* | MRA_3933 | ✔ | ✔ |  |
|  |  | *eccD2* | MRA_3926 |  | ✔ |  |
|  |  | *eccE2* | MRA_3924 | ✔ | ✔ |  |
|  |  | *espG2* | MRA_3928 |  | ✔ |  |
|  |  | *esxD* | MRA_3930 | ✔ | ✔ |  |
|  | ESX-3 (T7SS) | *PPE4* | MRA_0295 | ✔ | ✔ | STOP_GAIN |
|  |  | *eccA3* | MRA_0291 | ✔ |  |  |
|  |  | *eccC3* | MRA_0293 | ✔ | ✔ |  |
|  |  | *eccD3* | MRA_0299 |  | ✔ |  |
|  |  | *eccE3* | MRA_0301 |  | ✔ |  |
|  |  | *mycP3* | MRA_0300 |  | ✔ |  |
|  | ESX-4 (T7SS) | *eccB4* | MRA_3491 | ✔ |  |  |
|  |  | *eccC4* | MRA_3488 | ✔ | ✔ |  |
|  |  | *esxT* | MRA_3485 | ✔ | ✔ |  |
|  |  | *mycP4* | MRA_3490 | ✔ | ✔ |  |
|  | ESX-5 (T7SS) | *Undetermined* | MRA_1807 | ✔ | ✔ |  |
|  |  | *PE18* | MRA_1802 | ✔ |  |  |
|  |  | *PPE26* | MRA_1803 | ✔ |  |  |
|  |  | *PPE27* | MRA_1804 |  | ✔ |  |
|  |  | *PPE41* | MRA_2457 | ✔ | ✔ |  |
|  |  | *cyp143* | MRA_1799 | ✔ | ✔ |  |
|  |  | *eccA5* | MRA_1811 |  | ✔ |  |
|  |  | *eccB5* | MRA_1797 | ✔ | ✔ |  |
|  |  | *eccCb5* | MRA_1798 | ✔ | ✔ |  |
|  |  | *eccD5* | MRA_1808 |  | ✔ |  |
|  |  | *mycP5* | MRA_1809 |  | ✔ |  |
| **Stress adaptation** | AhpC | *ahpC* | MRA_2455 | ✔ |  |  |
|  | Cu | *sodC* | MRA_0437 | ✔ |  |  |
|  | Pore-forming protein | *ompA* | MRA_0906 |  | ✔ |  |
