# Supplementary figures and images for: Whole genome sequencing and comparative genomics of Mycobacterium orygis isolated from different animal hosts to identify specific diagnostic markers
Source: Front Cell Infect Microbiol. 2023 Dec 22;13:1302393. doi: 10.3389/fcimb.2023.1302393 (PMC10770871; doi:10.3389/fcimb.2023.1302393)

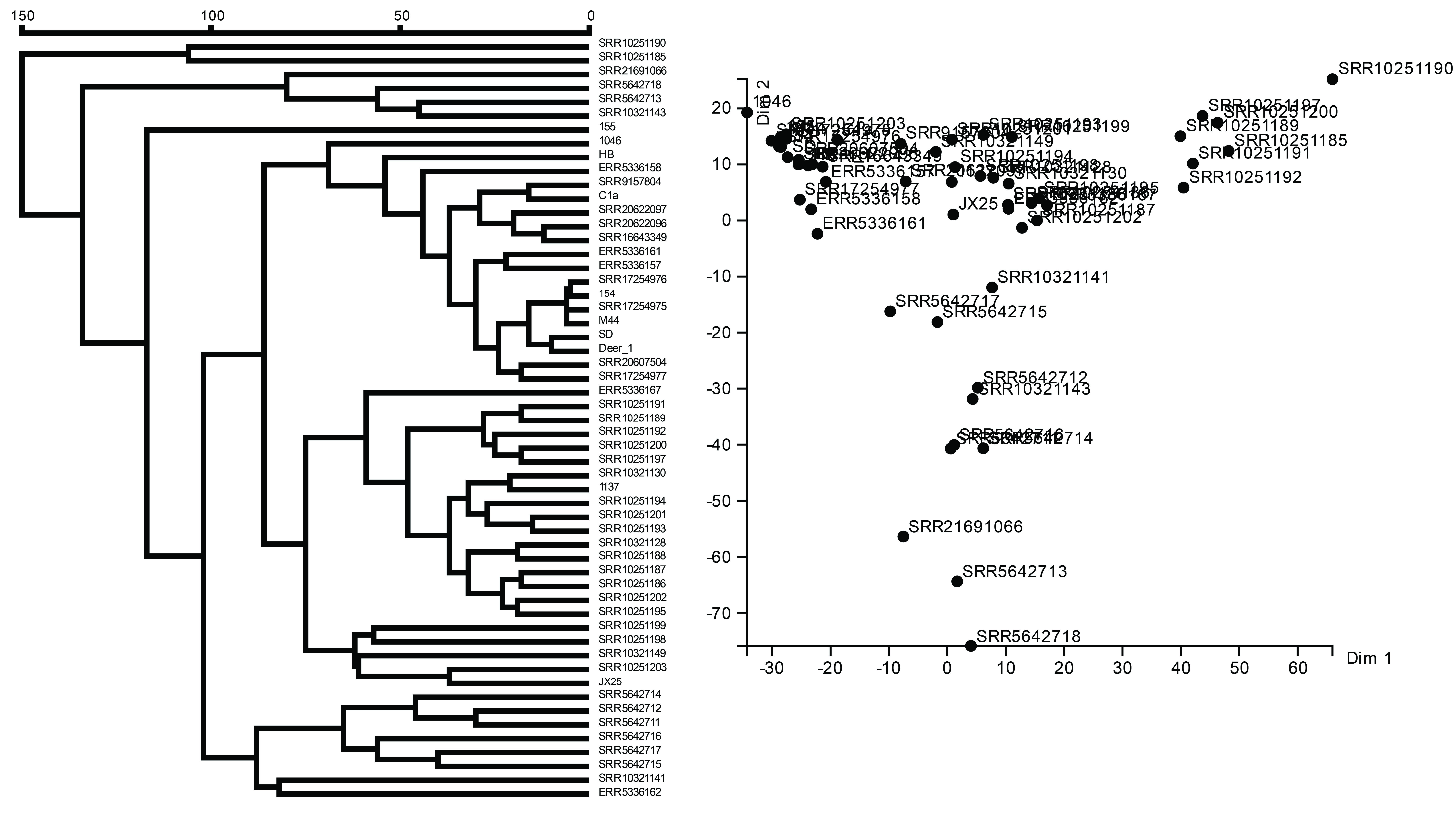

Supplement: Supplementary Figure 1 — Pangenome-based phylogeny and multidimensional scaling. A. Pangenome-based phylogeny. B. Multidimensional scaling of M. orygis genome. No major categorization based on place, year, or host of isolation. [file Image_1.jpeg]

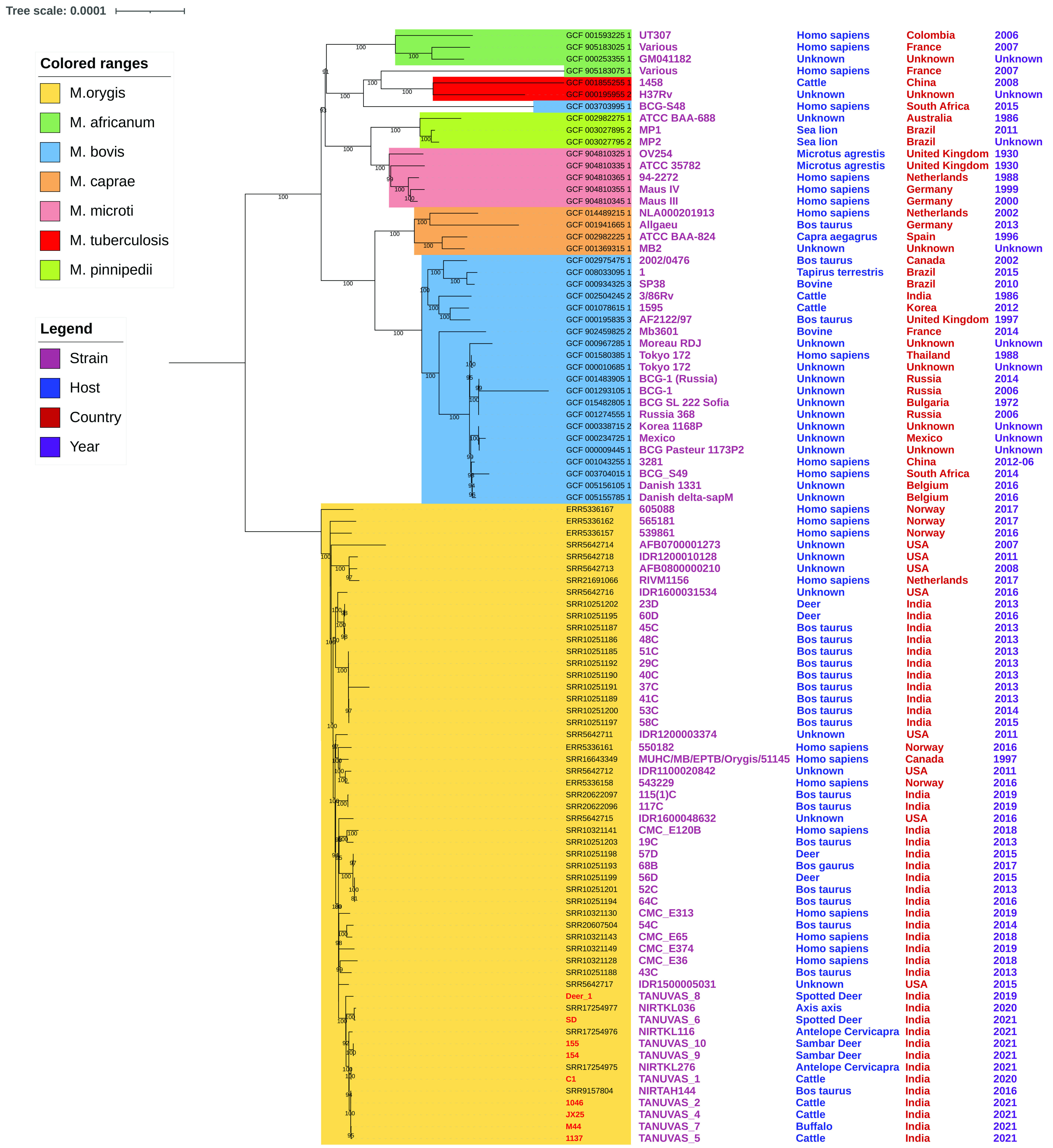

Supplement: Supplementary Figure 2 — Core genome phylogeny of MTBC isolates used in this study. [file Image_2.jpeg]

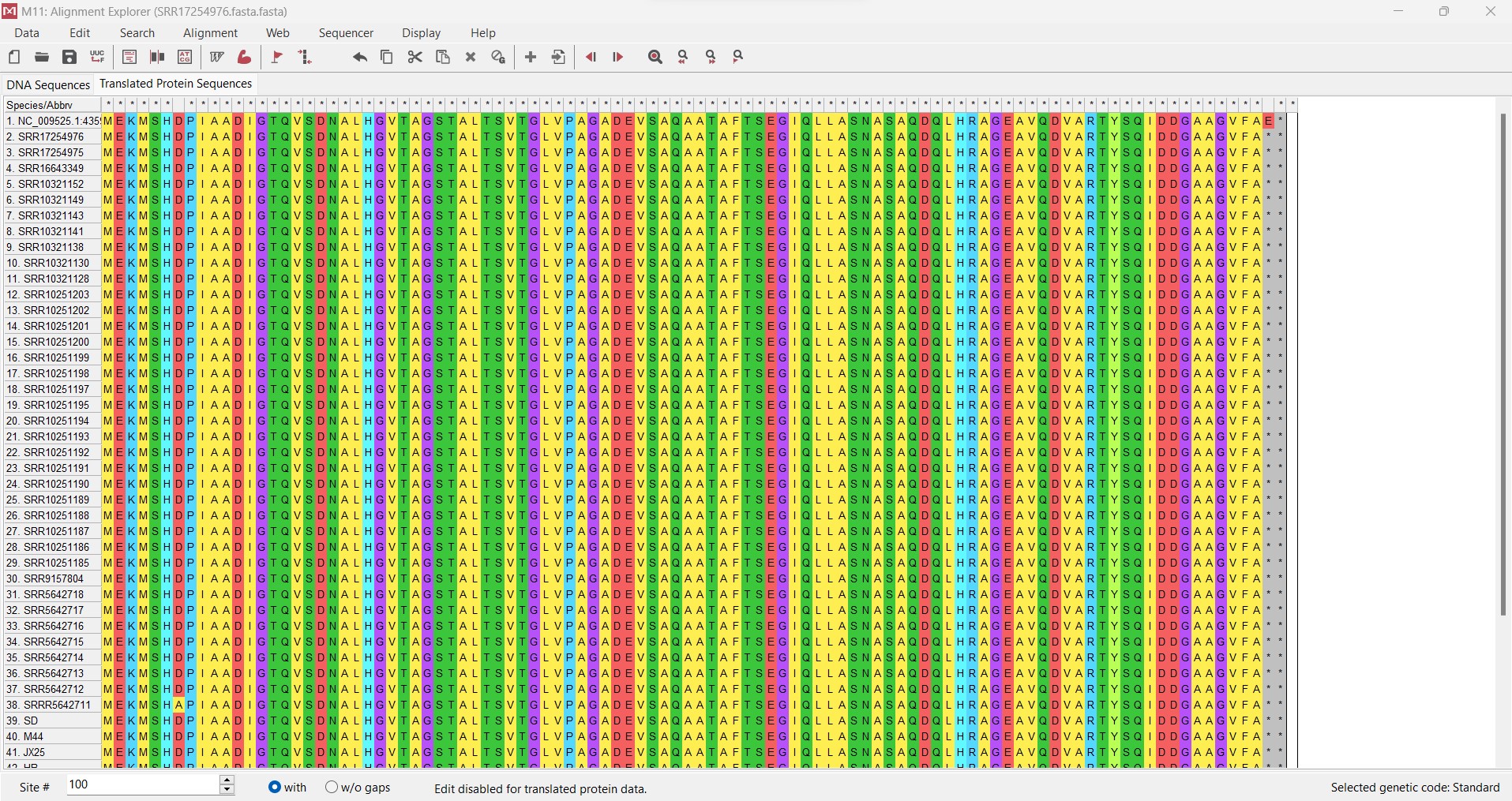

Supplement: Supplementary Figure 3 — Amino acid sequence alignment of PE35 gene showing stop codon (gained) marked as *. The first sequence is the M. tuberculosis reference sequence to which all the M. orygis is compared. [file Image_3.jpeg]

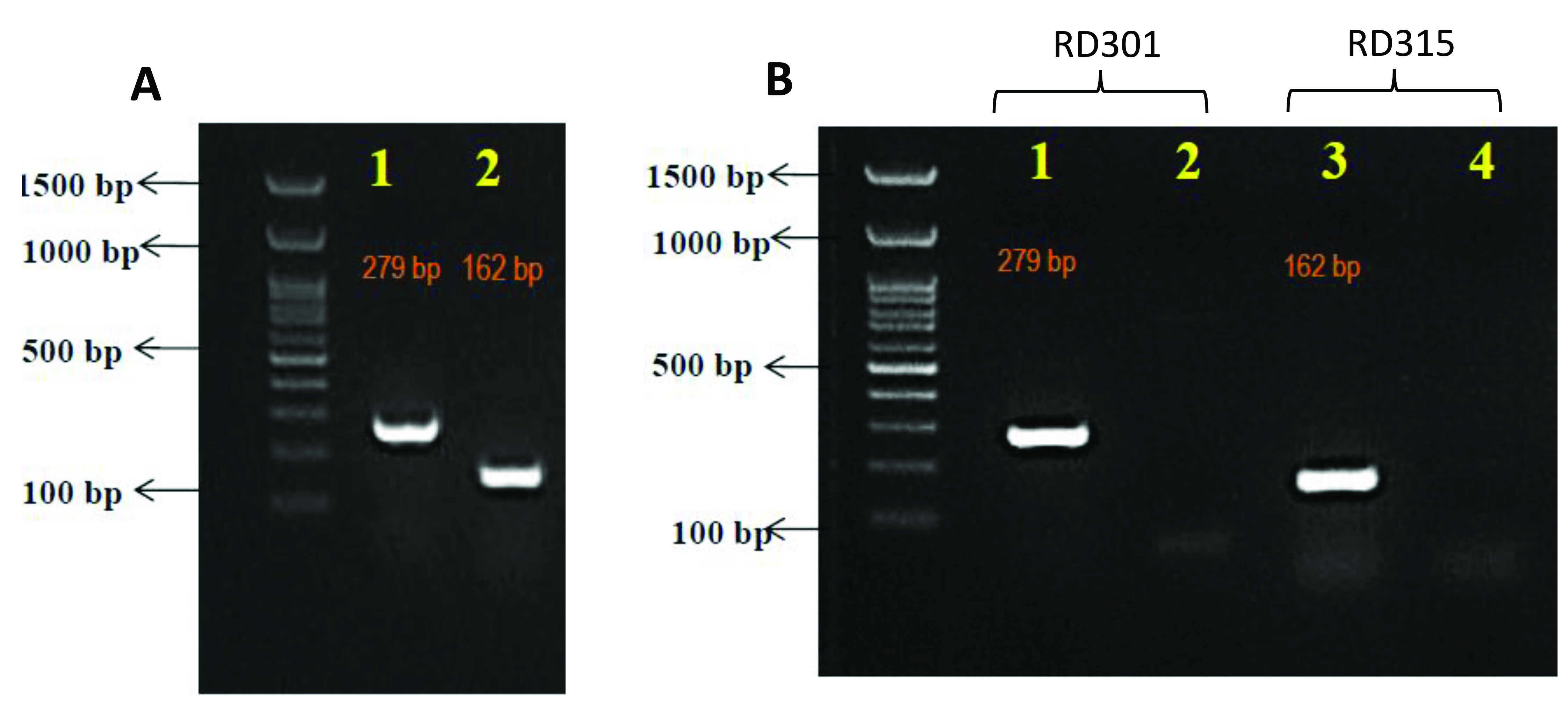

Supplement: Supplementary Figure 4 — Representative gel electrophoresis image of PCR for RD307 and RD315. A. M. tuberculosis DNA. Lane 1—RD301, Lane 2—RD315. B. M. bovis BCG and M. orygis DNA. Lanes 1 and 2—M. bovis BCG and M. orygis tested for RD301 region respectively. Lanes 3 and 4—M. bovis BCG and M. orygis tested for RD315 region respectively. [file Image_4.jpeg]
